# *VirtualPain*. Preliminary findings from a group-based digital therapeutics intervention for fibromyalgia

**DOI:** 10.1177/20494637231210391

**Published:** 2023-11-03

**Authors:** Ferran Vilalta-Abella, Bruno Porras-Garcia, Alexandra Ghiţă, Montserrat Vacas-Moreira, Mónica Prat-Galbany, José Gutiérrez-Maldonado

**Affiliations:** 1 Unit of Psychology, Department of Psychiatry, 223474 Hospital of Sant Joan Despí Moisès Broggi, Sant Joan Despí, Spain; 2 BrainXR Lab, Department of Basic Sciences, Universitat Internacional de Catalunya, Sant Cugat del Vallès, Spain; 3 Research Group on Brain, Cognition, and Behavior, Consorci Sanitari de Terrassa (CST), Terrassa, Spain; 4 Unit of Health, Medical and Neuropsychology, Leiden University, Leiden, the Netherlands; 5 Department of Clinical Psychology and Psychobiology, 207203 University of Barcelona, Barcelona, Spain

**Keywords:** cognitive-behavioral group therapy, digital therapeutics, fibromyalgia, group treatment, pain perception

## Abstract

**Introduction:**

Fibromyalgia (FM) is a disorder characterized by chronic pain, with significant medical, psychological, and socio-economic implications. Although there is limited evidence, cognitive-behavioral therapy (CBT) has shown to be effective in improving FM symptoms. An alternative to enhance CBT effectiveness is to incorporate digital therapeutics (DTx).

**Aim:**

We conducted a pilot study to investigate whether the addition of a DTx intervention (*VirtualPain*) to cognitive-behavioral group therapy (CBGT) can reduce pain perception and associated symptoms in patients with FM.

**Method:**

Ten patients with FM were initially recruited from a public hospital in Barcelona. The treatment consisted of 6 weeks of *VirtualPain* group sessions and 16 weeks of CBGT. Measures of catastrophizing, self-efficacy, and coping were recorded before, during, and after the protocol. In the DTx sessions, pain intensity was recorded before and after each session.

**Results:**

The program (DTx and CBGT) showed a significant improvement in pain-related self-efficacy and relaxation measures. Improvement in pain perception was observed only after the DTx intervention.

**Conclusions:**

This study provides preliminary results regarding the added value of DTx (*VirtualPain*) as part of a CBGT for FM. The use of the program has facilitated a significant reduction in pain perception in each of the *VirtualPain* sessions, which provides further evidence of how this technology can be beneficial for improving FM treatments.

## Introduction

Fibromyalgia (FM) is a chronic disorder characterized by musculoskeletal pain and other symptoms including paresthesia, sleep disturbances, mood changes, fatigue, and difficulty concentrating.^1–4^ The prevalence of FM is 2.4% in the general population, more common in adult women (4.2%) than in adult men (0.2%). The clinical manifestation of FM is chronic pain which can vary in intensity, accompanied by psychological mechanisms such as anxiety, low mood, cognitive styles like catastrophizing, low self-efficacy, and coping strategies.^5–9^

The pathophysiological mechanism of FM remains unsatisfactorily understood. A multimodal treatment including pharmacological intervention,^10,11^ physical exercise,^12–14^ and cognitive-behavioral treatment (CBT) is usually offered to patients with FM. CBT aims to improve pain management and associated psychological symptoms such as anxiety, distress, catastrophizing, low self-efficacy, depression, and sleep disturbances.^2,15–20^ CBT can be conducted individually or in a group format (cognitive-behavioral group therapy, CBGT). CBGT has provided a more efficient and affordable treatment for healthcare settings with high patient demand, while also showing consistent empirical evidence in several conditions with medical and psychological implications.^21–25^ Previous studies reported that group interventions for patients with FM achieve significant improvements, but the data remain inconsistent.^25,26^

Digital therapeutics (DTx) are digital tools (e.g., software solutions, or mHealth/eHealth applications) developed to address a medical/psychological conditions. There has been an increase in DTx use in the past years in the field of health, having a three-fold purpose, *prevention, management*, and/or *treatment*.^27^ DTx can complement conventional treatments by improving the quality of life of the individual. Such technology demonstrated its effectiveness in targeting various psychological and physical symptoms,^28–30^ including chronic and acute pain.^31–34^ DTx brings forward the added value of technology in terms of addressing the psychological treatment in FM. For instance, previous studies implemented 3D figures in software solutions to symbolize characteristics of acute pain with the ultimate goal of improving pain management.^35–39^

Our group has developed an affordable and easy-to-use 3D laptop version (*VirtualPain*)^39^ to help patients with FM visualize and modify different features of their experience of pain. The current study aims to provide further evidence regarding the efficacy of adding *VirtualPain* as part of CBGT in patients with FM. To address the objective of the study, the first hypothesis was that the program (DTx and CBGT) reduces levels of anxiety and pain perception in patients with FM. The second hypothesis was that the program augments levels of self-efficacy and coping strategies in patients with FM.

## Method

### Participants

Ten patients were initially recruited from the Moisés Broggi Hospital in Sant Joan Despí (Barcelona). The inclusion criteria were a formal diagnosis of FM made by a specialist in rheumatology, being over the age of 18, and sufficient Spanish proficiency. The diagnosis was determined considering the results of a clinical interview and meeting the International Statistical Classification of Diseases and Related Health Problems - 10th Revision (ICD-10) criteria for FM.^40–43^ Exclusion criteria were self-reported epilepsy,^44^ visual impairment, and metabolic disorders. Patients with severe mental health disorders (e.g., psychosis, bipolar disorder, major depressive disorder, addictions, etc.) were excluded from the study.

### Materials

#### Pre- and post-treatment measures digital therapeutics and cognitive-behavioral group therapy

*- Catastrophizing.* Catastrophizing cognitions related to pain perception were assessed using the Pain Catastrophizing Scale (PCS),^45,46^ which consists of 13 items and has a high internal consistency (*α =* 0.92).^47^
*- Self-efficacy.* The Chronic Pain Self-Efficacy Scale (CPSS),^48,49^ composed of 22 items, measures perceived self-efficacy and has a high internal consistency (*α* = 0.91). The CPSS can be divided into three factors with good internal consistency indices: self-efficacy for pain management (PSE) (*α* = 0.72), self-efficacy for coping with symptoms (CSE) (*α* = 0.85), and self-efficacy for physical functioning (FSE) (*α* = 0.98).^49^
*- Coping.* The Chronic Pain Coping Inventory (CPCI-42)^50,51^ contains 8 scales describing different pain coping strategies with good internal consistency indices: Guarding (*α* = 0.76), Resting (*α* = 0.73), Asking for help (*α* = 0.87), Relaxation (*α* = 0.76), Task persistence (*α* = 0.74), Exercise (*α* = 0.84), Seeking social support (*α* = 0.81), and Coping self-report (*α* = 0.80).^51^
- *Ecological momentary assessment of pain perception.* A Visual Analog Scale (VAS) was used to explore momentary levels of pain perception on a scale from 0 (no pain) to 10 (unbearable pain) during DTx exposure (*VirtualPain* software).^52^

### Software

**Figure 1.**
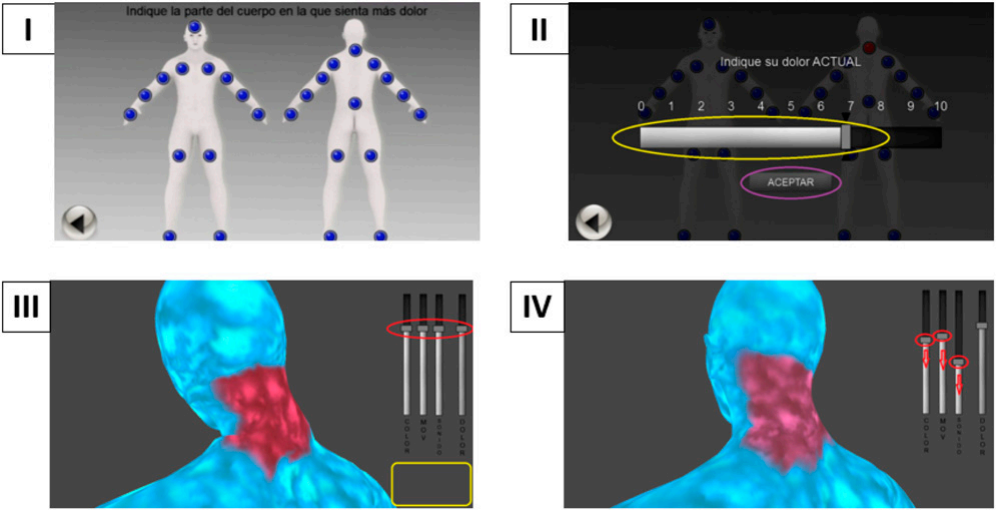
Software *VirtualPain*. *Note*: Sliding scales: color, movement, sound and pain intensity, respectively. Only the first three were used for the present study.

*VirtualPain* consists of a humanoid avatar divided into 27 body regions,^39,42^ as shown in Figure 1, Part I. The avatar is displayed in 3D, can be gender-personalized, and allows the user to individually select the most painful parts of their body. The software also has a built-in ecological momentary VAS from 0 (no pain) to 10 (unbearable pain) for each body area to assess pain intensity (Figure 1, Part II). In addition, the software allows pain representation through three features: color gradient, movement speed, and sound volume (Figure 1, parts III and IV). These parameters were implemented in our software based on previous studies reflecting pain experience of different body areas.^35,36,39^

### Hardware

Given the clinical symptoms of FM, it was decided to run *VirtualPain* on a semi-immersive three-dimensional (3D) laptop. The system included a hardware (3DStudio) that allowed the creation of a 3D stereoscopic effect, and polarized glasses with circular lenses. The *VirtualPain* screen was 15.6 inches and the participants stood 60 centimeters from the screen. A computer mouse was used to interact with the environment. In addition, headphones were used to increase immersion in the program.

### Procedure

#### Overview

This study was approved by the Ethics Committee of the University of Barcelona (*n*. IRB00003099) in collaboration with the Moisés Broggi Hospital in Sant Joan Despí (Barcelona). Patients from the Fibromyalgia Unit of the hospital (*N* = 50) who met the inclusion criteria were interviewed prior to enrollment; however, 10 patients agreed to participate in this study based on their written informed consent. Introductory meetings with the lead-researcher were conducted with the patients. The meetings consisted of informing the patients about the scope of the study, assessment tools and time points, the group format, and the length of the program. The program consisted of 22 sessions with a weekly frequency and a duration of 90 min/session. The intervention implied six DTx sessions (*VirtualPain* software), followed by 16 CBGT sessions.

**Figure 2.**
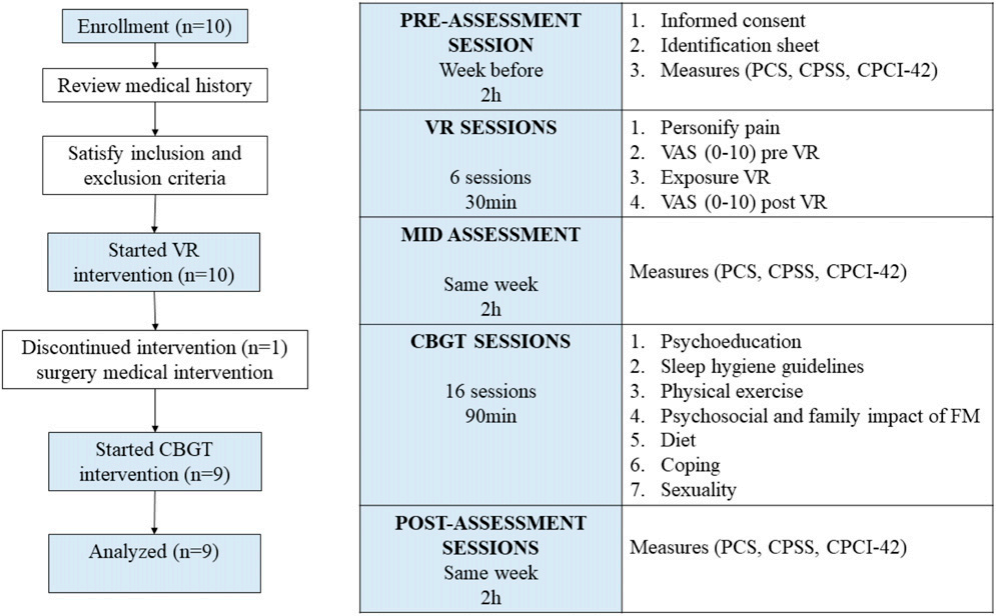
Participants’ journey through the pilot study.

As shown in Figure 2, the assessment time points were pre-intervention assessment session, after six sessions of *VirtualPain* (mid-assessment), and after 16 CBGT sessions (post-intervention assessment). During *VirtualPain*, momentary pain levels were assessed before and after the DTx session (Figure 2). Nine participants completed the study. One patient dropped-out of the study due to having an emergency surgery.

#### The *VirtualPain* – Cognitive-behavioral group therapy program

##### *VirtualPain* – Digital therapeutics group format

**Figure 3.**
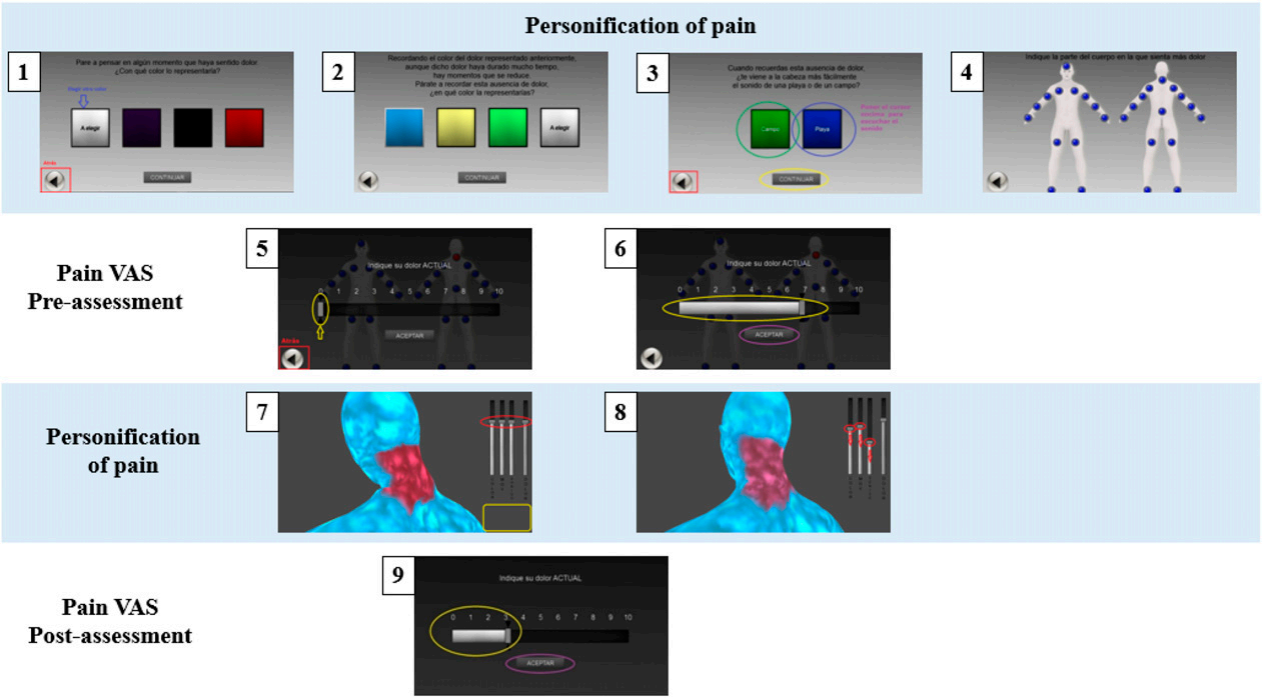
*VirtualPain* procedure. *Notes:* VAS = Visual Analog Scale.

The *VirtualPain* represents an embodiment of the perceived pain on one of the painful body parts. The software is aimed at exposing the patient to their painful body parts with the ultimate goal to reduce pain perception. The procedure of this DTx intervention is summarized in 9 steps (Figure 3), and divided into three phases: (a) pain personification (3-4 minutes), (b) pain quantification (1 minute), and (c) pain exposure (10 minutes).

- Personification: Patients embodied their painful body parts. They chose the (a) color that best represented their pain (step 1) and the (b) color of the absence of pain (step 2) and (c) sound (e.g., forest or beach) (step 3). Patients were asked to select one of the most painful body parts (out of 27 available body parts, which was step 4). Patients were exposed to a single body area per session. In the subsequent sessions, a similar procedure was followed for the same or other painful body areas.
- Quantification: Once the pain area had been selected by the patient, a VAS ranging from 0 (no pain) to 10 (unbearable pain) was displayed on the screen to quantify the intensity of pain perceived in that specific area (steps 5 and 6).
- Exposure: The software displayed a stereoscopic representation of the body area, colors, and sound chosen to symbolize the pain experience (step 7). Four sliding scales were displayed on the screen as previously selected by the patients in step 6 such as color gradient, speed of movement, volume of sound, and intensity of perceived pain.

The following therapeutic procedure was to encourage the patients to observe their painful body areas and gradually decrease the sliding scales related to the representation of pain. This exercise helped them to recognize and transfer this visual change to their own representation of pain (step 8). The progressive reduction of the pain-related sliding scales and the resulting visual change in the figure was intended to facilitate a modification of the patients’ representation of painful body areas. During the session they were encouraged to interact freely with the software to increase their autonomy and self-efficacy. The clinician-scientist only intervened when necessary for the correct functioning of the software. After 10 minutes of interaction, pain levels were assessed with the VAS (step 9). This procedure was repeated for the next five DTx sessions. The clinician-scientist played an increasingly passive role as each patient became more confident in interacting with the DTx software.

##### Cognitive-behavioral group therapy

The CBGT component of the program was delivered over the next 16 weeks. An experienced clinician-scientist from the hospital was responsible for delivering various CBT techniques commonly used in FM. A comprehensive overview of CBGT can be found in Table 1.

**Table 1.** Schedule (by sessions) of the applied techniques.

No. Session	Modules	Description
1–4	Psychoeducation FM	Explanation of FM, its symptoms,^53–57^ different effective treatments for FM: pharmacological, physical exercise and psychological intervention.^10,12,18^
5	Sleep hygiene guidelines	Sleep hygiene guidelines (e.g., healthy sleep patterns, fixed sleep schedule) were provided.^14,58,59^
6–7	Physical exercise	Promote healthy physical activity and challenge the negative automatic thoughts about exercise.^13,54,60,61^
8–11	Psychosocial factors of FM	Address the psychosocial (e.g., work, family, peers, leisure activities) of FM symptoms.^62,63^
12	Diet	Promote a healthy diet based on nutrients that boost muscle recovery after exercise.^64^
13–15	Coping	Promote active and adaptive coping strategies and increase perceived self-efficacy to cope with pain.^65,66^
14	Sexuality	Address sexual dysfunction, the perception of pain during intercourse and any associated problems with partners.^67^
16	Closing session	Program review and encouragement of future coping strategies in difficult situations.

*Notes:* FM = Fibromyalgia.

### Statistical analysis

A within-subjects design was depicted in this pilot study. Initial descriptive analyses were used to determine socio-demographic and other clinical measures assessed at baseline.

One-way repeated measures ANOVAs were used to determine whether statistically significant differences existed across the intervention (pre-/post-DTx and post-CBGT) in the primary measures (PCS, CPSS, and CPCI-42). Considering the small sample size and data normality assumptions, non-parametric tests were further applied. Wilcoxon signed-rank analyses were performed for the pain levels assessed before and after each DTx session. Friedman tests were performed to explore measurements at the three time points (pre-DTx, post-DTx, and post-CBGT). In the latter analyses, pairwise comparisons were performed with a Bonferroni correction for post-hoc analyses.

## Results

### Descriptive results

The mean age of our participants was 55.78 years (*SD* = 10.52, age range: 39 to 71 years), all participants identified themselves as females and reported being married. Other socio-demographic and medical history data of thepatients are shown in Table 2.

**Table 2.** Quantitative and qualitative sample variables.

Quantitative variables	*M*	*SD*
Age (years)	55.78	10.52
Dx FM (years)	42.78	10.23
Prescription drugs	4	1.94

Qualitative variables	
Employment status	%
Employed	22.2
Sick leave	11.1
Disability	33.3
Retired	11.1
Unemployment	22.2
Comorbidities	%
MS/CFS	55.6
MCS	11.1
IBS	22.2
Arthrosis	66.7
Migraine	44.4
Prolapsed disc	22.2
Spondylosis	33.3
Anxiety	88.9
Dysthymia	66.7
Biological sex	%
Female	100
Relationship status	%
Married	100
Medications	%
Pain	88.9
NSAID	33.3
Corticosteroids	22.2
Anxiolytics	55.6
Antidepressants	66.7
Antipsychotics	11.1

*Note*. Dx FM = diagnosis of FM; IBS = irritable bowel syndrome; MS/CFS = myalgic encephalomyelitis/chronic fatigue syndrome; MCS = multiple chemical syndrome; NSAID = nonsteroidal anti-inflammatory drugs.

#### Within-DTx intervention outcomes. Pain visual analog scale (VAS)

A Wilcoxon signed-rank test indicated significant reduction across all six *VirtualPain* sessions (Table 3) as determined by assessment prior and after each DTx sessions (*p* < .05).

**Table 3.** Measurements before and after the administration of the *VirtualPain*.

	VAS pre	VAS post-DTx	*z*	*p*
	Median	Median		
Session 1	8.02	5.12	−2.666	.008
Session 2	8.02	6.17	−2.429	.015
Session 3	8.04	5.05	−2.666	.008
Session 4	7.10	5.71	−2.521	.012
Session 5	7.22	5.60	−2.666	.008
Session 6	7.98	5.24	−2.666	.008

*Note*: VAS = Visual Analog Scale.

#### *VirtualPain* versus CBGT. Catastrophism, self-efficacy, and pain coping strategies

The results obtained before the intervention, after the 6 DTx group sessions, and after 16 CBGT sessions are summarized in Table 4. Regarding catastrophism, despite the data showed a gradual tendency of reduced scores, Friedman test analyses (PSC scale) were not statistically significant (*χ*^2^ (2) =3.829, *p* = .147).

**Table 4.** Repeated measures of anxiety, self-efficacy, and coping scales.

	Median pre	Median post-DTx	Median post-CBGT	*Chi*	*p*
PCS_Total	33	32	21	3.829	0.147
CPSS					
PSE	17	24	28	7.943	0.019*
FSE	33	36	43	4.667	0.097
CSE	24	31	49	6.686	0.035*
Total	68	84	120	6.889	0.032*
CPCI					
Coping	28	27	39	2.889	0.236
Exercise	42	50	51	1.471	0.479
Persistence	21	22	20	2.294	0.318
Social	9	14	11	2.529	0.282
Assistance	27	22	23	3.486	0.175
Resting	43	45	42	3.879	0.144
Relaxation	8	14	14	8.706	0.013*
Guarding	28	26	24	0.743	0.690

*Note*. PCS Total: Pain Catastrophizing Scale; CPSS PSE=Pain Management Self-Efficacy; CPSS FSE=Physical Function Self-Efficacy; CPSS CSE=Symptom Coping Self-Efficacy; CPSS Total=Chronic Pain Self-Efficacy Scale; CPCI = Chronic Pain Coping Inventory. *p < .05.

Regarding self-efficacy (CPSS scale), Friedman’s test analyses showed statistically significant differences at different assessment times, in the CPSS total score (*χ*^2^ (2) =6.889, *p* = .03), the PSE subscale (*χ*^2^ (2) =7.943, *p* = .02), and the CSE subscale (*χ*^2^ (2) =6.686, *p* = .03). On the CSE subscale, post-hoc analyses indicated a significant improvement between pre-DTx and post-CBGT scores (*mdn* Pre-DTx= 24, *mdn* Post-CBGT = 49, *p* = .04). There were no statistically significant improvements before and after *VirtualPain*/CBGT interventions. On the PSE subscale, post-hoc analyses showed a significant improvement in scores before and after the CBGT intervention (*mdn* PostDTx= 24, *mdn* Post-CBGT = 28, *p* = .02). No statistically significant differences were observed pre- and post-DTx intervention.

Regarding pain coping strategies as assessed by the CPCI scale, Friedman’s test analyses showed statistically significant differences at the different assessment times only in the Relaxation subscale (*χ*^2^ (2) =8.706, *p* = .01). Post-hoc analyses revealed a statistically significant increase in relaxation subscale scores only before and after the DTx intervention (*mdn* Pre-DTx = 8, *mdn* PostDTx = 14, *p* = .029), whereas no statistically significant differences were observed pre- and post-CBGT.

## Discussion

This pilot study provides preliminary evidence regarding the added value of DTx in the treatment of FM. Two complementary interventions were proposed as part of a comprehensive psychological treatment for FM, *VirtualPain*, and CBGT, which consisted of 22 group sessions. The data of this program indicated significant improvements regarding pain perception, relaxation, and pain management self-efficacy in patients with FM. In addition, *VirtualPain* intervention was particularly helpful in improving pain perception and relaxation. These changes were maintained during CBGT.

The observed results on the relaxation measure may be explained by the relaxing stimuli included in the software (e.g., the landscape and the sound of the beach). Previous studies showed that exposure to digital nature environments augments relaxation and promotes well-being.^68,69^ Therefore, *VirtualPain* may be particularly suitable in facilitating relaxation. This process enhances coping mechanisms for pain and stress management.^70^

One of the most prominent hallmarks of this study is the significant reduction in pain perception after each DTx sessions. However, this change was not significant in other variables such as catastrophism, coping, or pain self-efficacy. These results align with a recent systematic review^71^ that identified the variable *efficacy* of psychological therapies in enhancing pain perception in FM.

Although a significant improvement in pain self-efficacy was expected, the *VirtualPain* intervention showed only a tendency. Nevertheless, a brief intervention with six DTx sessions may not be sufficient for patients to internalize new active coping strategies or to change their negative belief system about pain management.^70^

Regarding CBGT, there was a significant improvement in measures of self-efficacy for pain management (PSE). These findings are consistent with previous research that highlighted the usefulness of CBGT in providing FM patients with information, tools, and resources to improve their active coping with pain.^70^ In addition, CBGT was delivered over a longer period of time (i.e., 16 weeks), in contrast to the *VirtualPain* intervention, which may facilitate a more profound change in their negative pain beliefs and coping strategies.

Lastly, no significant improvements were observed in other variables such as physical activity, social support, and sleep quality. These results are in line with previous studies showing inconsistent results in the application of CBGT to improve these secondary measures of pain in patients with FM.^25,26,72^

DTx represent an added value to support professionals in complementing and enhancing the traditional treatment approaches. Such digital tools are at the beginning of its development in the field of FM, but ongoing research emphasizes the opportunities for additional management and intervention tools.^73,74^

*VirtualPain* is a new technological device that represents an innovative DTx approach to enable patients with FM to cope with pain more actively. The physical representation of pain is an abstract phenomenon, without shape or tangible characteristics. However, *VirtualPain* allowed patients to represent pain shape, color, movement, and sound, while accurately localizing it to specific areas of the avatar’s body. Since a physical embodiment of pain was available involving multiple sensory inputs (visual and auditory), *VirtualPain* promoted relaxation and a better coping with pain.^75,76^ *VirtualPain* has a very intuitive and easy to use interface, which allowed the clinician-scientists to take a more passive and supervisory role during the intervention. This could be a potential advantage when offering *VirtualPain* to patients remotely (but under supervision) in other non-hospital settings (e.g., at home) as an in-between session support. Considering the high cost of FM treatment in healthcare settings,^77^ DTx may be a complementary tool to reduce the cost of psychological interventions.^78^ *VirtualPain* may be a suitable adjunct to psychological treatment for FM which does not require costly ongoing health care.

This pilot study has important methodological limitations related to the small sample size (i.e., only 9 patients completed the study) and the lack of an active control condition. In the coming years, we expect to conduct a randomized clinical trial with an active control condition to specifically evaluate the influence of *VirtualPain* in CBGT delivered to patients with FM. Another important limitation is the lack of mid- and long-term follow-up measures to assess long-term improvements. Considering the fact that pain levels measured on VAS returned to their baseline levels after each session, it would also be interesting to increase the number of DTx sessions to assess whether longer exposure will promote the development of active pain coping strategies in patients with FM. *VirtualPain* will also benefit from increasing body areas and adding further input stimuli (visual, auditory, and haptic).

In conclusion, this study presents preliminary results about the added value of *VirtualPain* to CBGT. The DTx intervention significantly reduced the perception of pain. The data suggest *VirtualPain* is an attractive and potentially beneficial complementary intervention to standard treatment for FM. DTx provides an opportunity to reduce the cost of FM treatment of the healthcare system.
